# 

**Published:** 1917-04

**Authors:** 


					﻿Voi.xXxn
APRIL, 1917
No. 10
TEXAS
Medical Journal
				

